# The role of patients and healthcare workers *Staphylococcus aureus* nasal colonization in occurrence of surgical site infection among patients admitted in two centers in Tanzania

**DOI:** 10.1186/s13756-019-0554-y

**Published:** 2019-06-17

**Authors:** Nyambura Moremi, Heike Claus, Ulrich Vogel, Stephen E. Mshana

**Affiliations:** 1 Institute for Hygiene and Microbiology, University of Wuerzburg, Wuerzburg, Germany; 2 Department of Microbiology and Immunology, Catholic University of Health and Allied Sciences, P. O. Box 1464, Mwanza, Tanzania

**Keywords:** *S. aureus*, Colonization, Surgical site infection, Tanzania

## Abstract

**Background:**

Colonization with *Staphylococcus aureus* has been identified as a risk for subsequent occurrence of infection. This study investigated the relationship between *S. aureus* colonization of patients and healthcare workers (HCWs), and subsequent surgical site infections (SSI).

**Methods:**

Between December 2014 and September 2015, a total of 930 patients and 143 HCWs were enrolled from the Bugando Medical Centre and Sekou Toure hospital in Mwanza, Tanzania. On admission and discharge nasal swabs, with an additional of wound swab for those who developed SSI were collected from patients whereas HCWs were swabbed once. Identification and antimicrobial susceptibility testing were done by VITEK-MS and VITEK-2, respectively. Detection of Panton Valentine leukocidin (PVL) and *mec*A genes was done by PCR. *S. aureus* isolates were further characterized by *spa* typing and Multi-Locus Sequence Typing (MLST).

**Results:**

Among 930 patients screened for *S. aureus* on admission, 129 (13.9%) were positive of which 5.4% (7/129) were methicillin-resistant *S. aureus* (MRSA). Amongst 363 patients rescreened on discharge, 301 patients had been tested negative on admission of whom 29 (9.6%) turned positive after their hospital stay. Three (10.3%) of the 29 acquired *S. aureus* were MRSA. Inducible Clindamycin resistance occurred more often among acquired *S. aureus* isolates than among isolates from admission [34.5% (10/29) vs. 17.1% (22/129), *P* = 0.018]. *S. aureus* contributed to 21.1% (*n* = 12) of the 57 cases of investigated SSIs among 536 patients followed. Seven out of eight *S. aureus* carriage/infection pairs had the same *spa* and sequence types. The previously reported dominant PVL-positive ST88 MRSA strain with *spa* type t690 was detected in patients and HCW.

**Conclusion:**

A significant proportion of patients acquired *S. aureus* during hospitalization. The finding of more than 90% of *S. aureus* SSI to be of endogenous source underscores the need of improving infection prevention and control measures including screening and decolonization of high risk patients.

## Introduction

*Staphylococcus aureus* is a human commensal found most commonly in the anterior nares and in other extra-nasal sites such as the perineum, skin, pharynx and in small proportion in the gastrointestinal tract and axillae [1–4]. About 20% of the population is reported to be persistent nasal carriers of *S. aureus*, 60% are intermittent carriers and about 20% are non-carriers [5]. *S. aureus* has been implicated in a number of clinical infections ranging from minor skin and soft tissue infections to life threatening conditions such as pneumonia and toxic shock syndrome [6].

Colonization of patients with *S. aureus* has been linked to the occurrence of subsequent infections [6]. Under the umbrella of exogenous sources of healthcare-associated infections (HCAIs), healthcare workers (HCWs) have been identified as reservoirs and potential niche for cross-transmission of HCAIs causing pathogens [7]. Colonization and acquisition of methicillin-resistant *S. aureus* (MRSA) during hospital stay not only complicate the management of patients but also significantly affects efforts to prevent and control HCAIs [8].

In Tanzania, surgical site infection (SSI) rates range from 10.9 to 26% [9–12] and *S. aureus* is the most isolated pathogen. Despite the predominance of *S. aureus* SSI in Tanzania, the link of patients’ colonization and the role of HCWs colonization as possible reservoirs of *S. aureus* have not yet been established. The current study was done to investigate the role of pre-and post-admission *S. aureus* colonization among surgical patients in subsequent occurrence of SSI. In parallel, HCWs attending patients in the same surgical wards were also screened for *S. aureus* nasal colonization to look for evidence of carriers who might act as reservoir and serve as a potential source of *S. aureus* cross transmission.

## Methods

### Study design and site

It was a prospective cohort study conducted for a period of 10 months (December 2014–September 2015) at the Bugando Medical Centre (BMC) and Sekou Toure hospital. Sekou Toure is a regional hospital of Mwanza, it has a bed capacity of 300 and well-established operating theatres. BMC has a bed capacity of 1000 and it serves as a zonal referral hospital caring for about 18 million people residing in 10 out of 30 regions of Tanzania. BMC is also a teaching hospital for the Catholic University of Health and Allied Sciences (CUHAS).

### Sample size estimation of the SSI survey

Kish’s formula (1965) was used to estimate the sample size assuming an average prevalence of 18.5% from the two short clinical studies on SSI done in Mwanza city in 2010 [12] and 2012 [10] whereby a minimum of 230 patients were to be enrolled. However, considering the design effect of the two sites, the estimated sample size was inflated by 10% which added to 253 as the minimum sample size.

### Patients and HCWs

Consented patients admitted for surgery between December 2014 and September 2015 were enrolled into the study. Patients’ social-demographic information and medical history relevant to the study were recorded using a data collection tool. Patients’ nasal swabs were taken before surgery within 48 h of admission to assess *S. aureus* carriage status before hospital stay. When patients were discharged from the hospital, a second nasal swab was taken to assess *S. aureus* carriage status during hospital stay (*S. aureus* acquisition). After surgery, patients were followed up by either a surveillance doctor or a trained nurse to observe the signs of infection at the incision site while in the wards. In case of SSI development pus swabs were taken for microbiological investigations [13]. Discharged patients were followed-up using their mobile phone numbers until either a SSI became apparent or up to 30 days after being operated. Some of the patients whose surgeries involved foreign body implantation were followed-up for 90 days [13].

In parallel, consented HCWs working in surgical wards were also enrolled. A nasal swab was taken only once to assess their *S. aureus* carriage status.

### Sample collection, culture and antimicrobial susceptibility testing

For nasal swabs, sterile cotton tipped swab (Mast Group Ltd., United Kingdom) was moistened in 0.85% sterile saline before swabbing, and then introduced into one nostril (nasal vestibule) to a maximum of 1.5 cm (range swab). The swab was rotated three times around one inner nasal circumference and the same swab was introduced into the second nostril, and the same was repeated as in the first nostril. The swab was inserted into Amies transport medium (Mast Group Ltd., United Kingdom) and transported to the Catholic University of Health and Allied Sciences (CUHAS) laboratory within two hours for processing. For wound infections, the surface of the infected surgical site was first cleaned with normal saline before swabbing [14].

All nasal and wound swabs were inoculated onto Columbia blood agar (OXOID LTD, Basingstoke, Hampshire, England) for *S. aureus* isolation. The plates were incubated aerobically at 37 °C and examined for growth after 18–24 h. Preliminary *S. aureus* screening was based on colony morphology i.e. either yellow to golden yellow or creamy-white colonies with or without beta-hemolysis on blood agar [15]. Colony morphology identification was followed by gram-staining, catalase, slide coagulase and DNase tests.

All positive *S. aureus* isolates were further confirmed by VITEK-MS system (bioMérieux, France).

The antimicrobial susceptibility testing was done using automated VITEK-2 system (bioMérieux, France) following manufacturer’s instructions. Briefly, a standardized inoculum with 0.5 McFarland-standard was made from pure culture. The test tube with the inoculum was placed into the VITEK-2 cassettes at the Smart Carrier Station™ to virtually link the sample and the AST-GP632 (VITEK-2 Card specific for *S. aureus*). Cefoxitin test for MRSA was performed by Kirby disc diffusion method on Mueller-Hinton E- Agar plates (bioMérieux, France). Isolates with zone of inhibition ≤21 mm were phenotypically considered as MRSA and [16] were confirmed by presence of *mec*A gene.

### Amplification of PVL and *mec*A genes

Moremi et.al [17], The PVL gene was amplified using *luk*SF-Forward (5′-ATCATTAGGTAAAATGTCTGGACATGATCCA-3′) and *luk*SF-Reverse (5′-GCATCAAGTGTATTGGATAGCAAAAGC-3′), whereas *mec*A1-Forward (5’GTAGAAATGACTGAACGTCCGATAA-3′) and *mec*A2-Reverse (5′-CCAATTCCACATTGTTTCGGTCTAA-3′) primers were used to amplify mecA gene. The known PVL-positive MRSA isolate VA17763 (spa type t008) was used as a positive control. The PCR reaction was carried out in a 50-μl volume mixture which included 400 nM of each primer, 1X buffer II, 200 μM of each deoxynucleoside triphosphate, 1.5 mM MgCl_2_, and 2 U of AmpliTaq Gold DNA polymerase (Thermo Scientific™, Darmstadt, Germany) and a 4 μl (OD_600_ = 0,1) DNA template.

### *Spa* typing and multilocus sequence typing (MLST)

Spa typing and MLST was employed to 60 selected *S. aureus* isolates of which 58 were from patients and two were from HCWs. The selection of the 60 *S. aureus* isolates for typing was based on either they are carriage and infection pairs, isolates from other patients (the same cohort in the same ward) admitted either 2 weeks before or after the occurrence of SSI of patients, as well as all MRSA isolates from patients and HCWs.

*Spa* typing was performed using the primers 1095-Forward (5′-AGACGATCCTTCGGTGA-3′) and 1517-Reverse (5′-GCTTTTGCAATGTCATTTACTG-3′) [18]. The PCR conditions for the primers were; initial denaturation at 95 °C for 10 min, 30 cycles were run with 94°for 30s, annealing at 55°for 30s and extension at 72 °C for 30s each, followed by a final extension step for 10 min at 72 °C. The PCR products’ purification and sequencing were commercially done at Microsynth SeqLab GmbH (Goettingen, Germany). Isolates were assigned to particular *spa* types using the spa server (http://www.spaserver.ridom.de, Ridom GmbH, Muenster, Germany).

The seven housekeeping genes of the respective bacterial isolates were amplified as per previously described protocol [19]. TraceEdit Pro software (Ridom GmbH, Germany) and SeqMan Pro software (DNASTAR Inc. USA) were used to trim, quality-check and analyze the sequences. Isolates were assigned to their respective sequence types according to MLST website https://pubmlst.org/saureus.

### Data analysis

Data were entered in Microsoft Excel 2010 and analyzed using STATA-13 software (STATA Corp LP, USA). Categorical variables such as history of admission, history of surgery, history of antibiotic use etc., were presented as proportions and compared using chi-squared test. Continuous variables such as age and duration of hospital stay were presented as medians [inter quartile range (IQR)] and compared using the Wilcoxon-Mann-Whitney test. To identify factors associated with *S. aureus* carriage univariate regression followed by backward multivariate logistic regression analysis was done. A *P*-value of less than 0.05 was considered statistically significant.

### Ethical approval and consent to participate

The protocol for this study was reviewed and approved by the Joint Catholic University of Health and Allied Sciences/ Bugando Medical Centre ethics and scientific review committee (CREC/019/2014). All study participants signed an informed written consent.

## Results

### Patients’ and HCWs *S. aureus* nasal carriage and antimicrobial susceptibility pattern

Of the 930 patients screened on admission, 129 (13.9%) were colonized with *S. aureus* of which 5.4% (7/129) were MRSA. On discharge 363 patients were re-screened of whom 60 (16.5%) were colonized with *S. aureus*. Among these 29 out of 301 (9.6%) patients who had been tested negative on admission turned positive during their hospital stay. Three (10.3%) of the 29 acquired *S. aureus* were MRSA.

A total of 143 HCWs working in surgical wards of BMC and Sekou Toure hospital were screened for nasal *S. aureus* carriage of whom 11 (7.7%) were positive. Two (1.4%) of the 143 HCWs were colonized with MRSA.

All *S. aureus* isolates from patients were susceptible to fusidic acid, linezolid, mupirocin, teicoplanin and vancomycin. Overall, hospital-acquired *S. aureus* isolates were more resistant to commonly used antimicrobials than isolates from admission with significantly higher rates for erythromycin, rifampicin and tetracycline (Table 1). Inducible clindamycin resistance (ICR) occurred more in MRSA isolates than in methicillin-sensitive *S. aureus* (MSSA) isolates [17.3% (31/179) vs. 60% (6/10), *P* = 0.001]. ICR also occurred more often among acquired *S. aureus* isolates than in isolates from admission [34.5% (10/29) vs. 17.1% (22/129), *P* = 0.018].

**Table 1:** Resistance rates of *S. aureus* isolates on admission and discharge

Antimicrobial agent	Admission (*N* = 129) *n* (%)	Acquired (*N* = 29) *n* (%)	*P* value
Benzylpenicillin	124 (96.1)	29 (100)	0.139
Clindamycin	2 (1.6)	1 (3.4)	0.254
Daptomycin	2 (1.6)	0	0.750
Erythromycin	32 (24.8)	12 (41.4)	0.035
Fusidic acid	0	0	–
Gentamicin	5 (3.9)	1 (3.4)	0.548
ICR^a^	22 (17.1)	10 (34.5)	0.018
Levofloxacin	1 (0.8)	1 (3.4)	0.126
Linezolid	0	0	–
Mupirocin	0	0	–
Rifampicin	9 (6.9)	7 (24.1)	0.003
Trimethoprim-sulfamethoxazole	68 (52.7)	19 (65.5)	0.105
Tetracycline	26 (20.2)	14 (48.3)	0.001
Teicoplanin	0	0	–
Vancomycin	0	0	–

^a^ *ICR* Inducible clindamycin resistance positive

### *S. aureus* SSI and associated factors

Of the 536 followed-up patients, 78 (14.6%) developed SSI. In addition, out of 57 patients with SSI who were available for microbiological investigations, 12 (21.1%) cases were due to *S. aureus* of which two were infected with MRSA. The other 78.9% of SSI cases were due to varieties of gram-negative bacteria as detailed in previous publications [20, 21]. No association was found between *S. aureus* SSI and colonization status, history of previous hospitalization, use of antibiotics, history of previous surgeries and history of chronic diseases.

### Sequence type (ST) distributions, *spa* types and PVL genes

MLST was employed to 60 *S. aureus* (58 from patients i.e. 12 SSI isolates and 46 carriage isolates, and two carriage isolates from HCWs). Of the 46 patients’ carriage isolates typed, 17 were from admission carriage and 29 were from discharge (13 acquired). Overall ST distribution was diverse in both groups of isolates. Eight STs were detected in the 17 isolates from admission of which ST8 (4/17) and ST5 (4/17) were dominant. Nine STs were detected among 13 acquired isolates typed of which ST152 (3/13) and ST5 (2/13) were predominant (Table 2).

**Table 2:** *S. aureus* sequence type (ST) distribution among admission, hospital acquired and surgical site infection (SSI) isolates

ST	Admission (*N* = 17)	Acquired (*N* = 13)	SSI (*N* = 12)
8	4	1	–
5	4	2	2
152	3	3	3
3118	2	1	1
72	1	2	1
88	1	1	1
4224^a^	1	–	–
4268^a^	1	–	–
188	–	1	–
508	–	1	–
707	–	1	–
612	–	–	2
22	–	–	1
4266*a	–	–	1

^a^New ST

Regarding the 12 *S. aureus* SSI isolates, eight STs were detected. Identified STs were ST152 (3), ST5 (2), ST612 (2), ST22 (1), ST72 (1), ST88 (1), ST3118 (1) and a new ST4266 (1). The two MRSA isolates from wounds were all typed as ST612. Out of eight *S. aureus* carriage/infection pair, seven pairs were clonal by bearing the same spa and ST. Three of the seven SSI pairs were due to carriage strains on admission, whereas the four cases of SSI were due to acquired strains (Table 3).

**Table 3:** Spa and MLST distribution among *S. aureus* carriage and SSI isolates

ID	Hospital	Ward	Region	Type of surgery	Spa^a^	ST^a^	Spa^b^	ST^b^	Spa-SSI	SSI-ST
4	Bugando	E8	Mara	ORIF	–	–	–	–	t690	88
26	Bugando	C6	Mara	Craniotomy	–	–	t4353	72	t4353	72
67	Bugando	C6	Shinyanga	Amputation	–	–	–	–	t355	152
106	Bugando	C6	Mwanza	Cholecystectomy	–	–	–	–	t355	152
150	Bugando	J5	Mara	CRelease	–	–	t095	508	t1257	612
152	Bugando	J5	Shinyanga	ORIF					t1257	612
191	Bugando	C9	Mara	VPShunt	t148	3118	t223	22	t223	22
379	Bugando	C6	Mwanza	Laparatomy	t355	152	t311	5	t311	5
ST111	SToure	MAT	Mwanza	Csection	t148	3118	t148	3118	t148	3118
ST148	SToure	MAT	Mwanza	Csection	t002	5	t223	4266*	t223	4266*
ST159	SToure	MAT	Mwanza	Csection	t002	5	t002	5	t002	5
ST302	SToure	MAT	Mwanza	Csection	t355	152	t355	152	t355	152

*a* admission, *b* discharge *SSI* Surgical site infection *CRelease* contracture release; *ORIF*; Open Reduction Internal Fixation; *Csection* Caesarean Section; *ST* sequence type; *New ST

Overall, ST88 was detected in six of all 60 MLST typed isolates of which five were PVL positive including one isolate from BMC HCW with *spa* type t690.

## Discussion

This study observed the admission and discharge *S. aureus* colonization rate to be 13.9 and 16.5%, respectively. The observed MRSA carriage of 5.4% on admission falls within a range of 0.2 to 7.2% observed among hospitalized patients in high-income countries [22–24]. The carriage rates are lower than 21% reported in Kwazul Natal, South Africa [25]. The difference observed in the two sub-Saharan African countries could be contributed by the nature of patients screened, in South Africa the majority of enrolled patients were HIV positive. HIV infection has been identified as a predictor of *S. aureus* colonization [26].

This study observed acquisition rate of 9.6 and 1% of *S. aureus* and MRSA, respectively. Although similar data are limited in African setting, the MRSA acquisition in this study is comparable to 1.7% previously reported in the United States [27]. Similar to the susceptibility patterns of *S. aureus* isolates reported in several African countries Tanzania inclusive [28], high rates of resistance to penicillin, trimethoprim-sulfamethoxazole and tetracycline were observed among carriage isolates in this study. Overuse and inappropriate prescription of these antimicrobials for various clinical conditions in Tanzania might be the driver for the observed high rates [29].

As observed previously in *S. aureus* isolates from BMC in Mwanza city [30] and those from a National hospital in Dar es Salaam city [31] in Tanzania, rate of ICR was significantly higher in MRSA than MSSA isolates. Similar findings have been reported among admitted patients in Tanzania [32] and Uganda [33]. Furthermore, as previously observed [34], the ICR rate was significantly higher in hospital-acquired than on admission *S. aureus* isolates. The fact that all SSI-MRSA isolates were hospital-acquired, and that 35% of acquired isolates were positive for ICR highlights a possibility of failure to treat *S. aureus* hospital-acquired infections empirically by clindamycin.

This study identified none of the isolates to be resistant to fusidic acid. This finding is within a 0–2% range of fusidic acid resistance rate observed in most sub-Saharan African countries [28]. The rare use of this antimicrobial in our setting could explain the observed findings.

The proportion of *S. aureus* causing SSI in the current study (21.1%) is within a range of 12–28.6% reported in different places in Tanzania including BMC where part of the current study took place [10, 12, 35]. The observation of more than 90% of *S. aureus* SSI in this study were of endogenous source can be explained by likely to occur nasal to wound transmission of *S. aureus* in case of poor hygiene [36].

The *S. aureus* STs 88, 5, 8 and 127 that were reported to be predominant in African continent were also detected in this study [37], including ST612 which was previously reported as a rare ST in South Africa [38]. These findings indicate the availability of specific *S. aureus* clones circulating in Africa.

Of note, the MRSA variant with *spa* type t7231 and ST1797 which was reported as predominant clone circulating in the same BMC surgical wards in 2010 [17] was not detected in the current study, suggesting either the possibility of outbreak in 2010 or change of *S. aureus* clones causing wound infections. Most of the ST88 detected were PVL positive as it was observed in BMC and other parts of the African continent [17, 28].

Although the *S. aureus* carriage rate of 7.7% observed among HCWs is significantly lower compared to 18.3% reported in Kenya [39], unlike in this study Kenya reported zero MRSA carriage. The difference might be due to the different nature of the study populations with HCWs of various low-risk cadres including physiotherapy in Kenya compared to high-risk nurses in this study who work in surgical wards with frequent exposure to septic/MRSA positive patients.

This study identified one HCW from BMC surgical ward who was colonized with a PVL positive MRSA ST88, previously reported to cause wound infections in the surgical wards of the same hospital [17]. The fact that this nurse has been serving the same surgical ward including when the previous study was carried out points to a possibility of HCWs to be reservoirs of MRSA strains causing infections in patients.

## Conclusion

Despite the large cohort, this study identified a low MRSA carriage among admitted patients and a low MRSA SSI. Most of the SSI were due to endogenous MSSA. Although the evidence is low, there is a possibility of HCWs being reservoirs of MRSA strains, a fact which highlights the role of screening them as part of the hospital IPC measures especially during outbreaks.

## Data Availability

All data have been included in this manuscript.

## Abbreviations

BMC: Bugando Medical Centre
CUHAS: Catholic University of Health and Allied Sciences
HCW: HealthCare Workers
MLST: Multilocus sequence
MRSA: Methicillin-resistant *S. aureus*
PVL: Panton Valentine leukocidin
SSI: Surgical site infection
ST: Sequence type
